# Cost-related medication nonadherence in Canada: a systematic review of prevalence, predictors, and clinical impact

**DOI:** 10.1186/s13643-020-01558-5

**Published:** 2021-01-06

**Authors:** Anne M. Holbrook, Mei Wang, Munil Lee, Zhiyuan Chen, Michael Garcia, Laura Nguyen, Angela Ford, Selina Manji, Michael R. Law

**Affiliations:** 1 Division of Clinical Pharmacology & Toxicology, Department of Medicine, McMaster University, Hamilton, ON Canada; 2 Department of Health Research Methods, Evidence and Impact, McMaster University, Hamilton, ON Canada; 3 Schulich School of Medicine & Dentistry, Western University, London, ON Canada; 4 Bachelor of Health Studies Program, University of Waterloo, Waterloo, ON Canada; 5 Bachelor of Health Sciences Program, McMaster University, Hamilton, ON Canada; 6 School of Medicine, Queen’s University, Kingston, ON Canada; 7 Global Health Program, McMaster University, Hamilton, ON Canada; 8 The Centre for Health Services and Policy Research, School of Population and Public Health, The University of British Columbia, Vancouver, BC Canada

**Keywords:** Medication adherence, Medication costs, Canada, Systematic review

## Abstract

**Background:**

Cost-related nonadherence to medications (CRNA) is common in many countries and thought to be associated with adverse outcomes. The characteristics of CRNA in Canada, with its patchwork coverage of increasingly expensive medications, are unclear.

**Objectives:**

Our objective in this systematic review was to summarize the literature evaluating CRNA in Canada in three domains: prevalence, predictors, and effect on clinical outcomes.

**Methods:**

We searched MEDLINE, Embase, Google Scholar, and the Cochrane Library from 1992 to December 2019 using search terms covering medication adherence, costs, and Canada. Eligible studies, without restriction on design, had to have original data on at least one of the three domains specifically for Canadian participants. Articles were identified and reviewed in duplicate. Risk of bias was assessed using design-specific tools.

**Results:**

Twenty-six studies of varying quality (*n* = 483,065 Canadians) were eligible for inclusion. Sixteen studies reported on the overall prevalence of CRNA, with population-based estimates ranging from 5.1 to 10.2%. Factors predicting CRNA included high out-of-pocket spending, low income or financial flexibility, lack of drug insurance, younger age, and poorer health. A single randomized trial of free essential medications with free delivery in Ontario improved adherence but did not find any change in clinical outcomes at 1 year.

**Conclusion:**

CRNA affects many Canadians. The estimated percentage depends on the sampling frame, the main predictors tend to be financial, and its association with clinical outcomes in Canada remains unproven.

**Supplementary Information:**

The online version contains supplementary material available at 10.1186/s13643-020-01558-5.

## Background

Medication cost-related nonadherence (CRNA) is defined as taking less medication than prescribed because of cost, such as delaying or failing to fill prescriptions, or skipping or lowering medication doses [1–3]. International estimates of the incidence and prevalence vary but are thought to be particularly high in the USA where many citizens are uninsured or under-insured [4–7]. Several factors have been found to be associated with nonadherence, including poor health, low household income, and disease burden [1, 8]. Cost-related factors proposed include lack of prescription drug coverage, high monthly medication cost, and high out-of-pocket costs [1, 8–12]. As for patient outcomes associated with CRNA, increased cost sharing was associated with the increased use of health services such as hospitalization and emergency department (ED) visits among patients with a number of chronic conditions [9, 13–15]. Treatment choices that patients at risk of CRNA face may lead to priorities that do not optimize health, such as choosing medications providing symptom relief only rather than important clinical benefit [16]. Other studies have suggested that higher medication adherence is associated with better outcomes and lower healthcare costs across many disease states and populations, including children [17–19]. However, all of these studies are susceptible to confounding due to their lower-quality design and the “healthy user effect”—the likelihood that adherent individuals have other unmeasured healthy behaviors [17]. Indeed, randomized trial evidence that removing financial barriers to essential medication access improves clinical outcomes is lacking. The landmark MI-FREEE trial showed that randomization to full coverage of key cardiac medications for patients post-myocardial infarction improved adherence but made no difference in the primary outcome of vascular events [20].

Although CRNA is well described in the USA and documented in other countries such as the UK and other European countries, it has not been as well characterized in Canada [21–23]. Total health expenditure in Canada was estimated to be $242 billion in 2017, with drugs accounting for 16.4% of the total and increasing at a faster rate than other sectors [24]. Furthermore, Canadians face some of the highest medication charges in the world, and while many individuals have private coverage, provincial-territorial public plans include some with very high co-pays and deductibles [25, 26]. Considering the effect that CRNA may have on patient outcomes and health care spending, knowledge of its prevalence, predictors, and clinical effects could help clinicians and policymakers to improve the effectiveness and cost-effectiveness of patient care. National PharmaCare themes under active discussion include national formulary creation, size, and reimbursement options [27, 28].

Given the current debate on medication costs, adherence, and PharmaCare policy nationally, we aimed to systematically review the literature to determine the prevalence, predictors, and clinical outcomes of CRNA in Canada. Our research question was “Amongst Canadians of any age, what is the prevalence of CRNA, what are its predictors using multivariable analysis, and what are the resultant clinical outcomes of CRNA?”

## Methods

This systematic review was designed in accordance with the most recent PRISMA statement (Additional file 1), but a review protocol was not registered [29, 30]. Eligible studies had to provide original data on at least one of the three stated objectives involving CRNA and Canadians. The following databases were searched since inception to the week of December 9, 2019: MEDLINE, Embase, Cochrane Library, and Google Scholar. The initial search terms used for MEDLINE and Embase were as follows: prescription fees, drug adj costs, exp patient compliance, medication adherence, cost sharing, health expenditures, and Canada/ or Canada. The Cochrane library search began with the terms “cost related adherence” and “Canada” and then limited, if needed, to include only studies involving Canada. For Google Scholar, the following searches were performed: “Cost-related nonadherence” and “Canada” combined with “medications” or “drugs” or “prescriptions.” No language restriction was applied. The authors of the key studies were surveyed for information on studies missed by our search or published since. The search strategy for MEDLINE is provided in Additional file 2.

Two authors screened the retrieved titles and abstracts. Articles were only included if they directly measured CRNA (i.e., not just adherence) prevalence. Studies examining the predictors of CRNA had to have used a multi-variable analysis that adjusted for multiple factors or measured differences in adherence in a randomized trial of an intervention directly targeting CRNA, or measured change in adherence immediately before and after a policy change where a change in patient costs or out-of-pocket expenses for medications is reasonably implicated. Studies examining the impact of CRNA were required to examine clinical outcomes such as hospitalization, adverse events, or disease. For example, self-reported increased health care utilization did not count. We included studies of any design without restriction on medication, age, sex, outcome, or measure of adherence. Studies were excluded if they did not report original data, were conference abstracts, or did not involve an identifiable Canadian population whose results were specified.

Articles passing through title and abstract screening underwent full-text screening then subsequent data extraction using pre-piloted forms. We extracted data on study design, sample size, CRNA definition, predictors, clinical outcomes, risk of bias, and statistical analysis. Two reviewers carried out duplicate full-text screening and data extraction independently, with differences resolved by consensus.

Risk of bias assessment was conducted using study design-specific tools. Surveys were rated on the representativeness of the sample, adequacy of response rate, missing data, pilot testing, and validity of the survey instrument, using a tool from Evidence Partners [31]. Qualitative studies were assessed using the Critical Appraisal Skills Program (CASP) checklist which asks about the appropriateness of qualitative design, recruitment, researcher-participant relationship, and data collection and analysis [32]. For pre-post studies, we assessed the intervention effect on the rate of outcomes over time, confounding, missing data, and selective reporting, using the Cochrane risk of bias criteria for interrupted time-series studies [33]. An overall risk of bias rating was calculated for each study based on the percentage of low risk of bias items (70–100% = low risk of bias, 31–69% = moderate risk, 0–30% = high risk). A summary risk of bias chart was created based on the Cochrane tool, showing each study as low, moderate, or high risk of bias [34].

Analyses planned included descriptive details of each study addressing at least one of our three components of CRNA, with additional focus on population-based studies (as opposed to disease- or drug-specific results). Quantitative data pooling of prevalence results was planned where permissible by the availability of compatible data, otherwise qualitative summaries of prevalence, predictors, and outcomes.

## Results

### Study characteristics

**Fig. 1:**
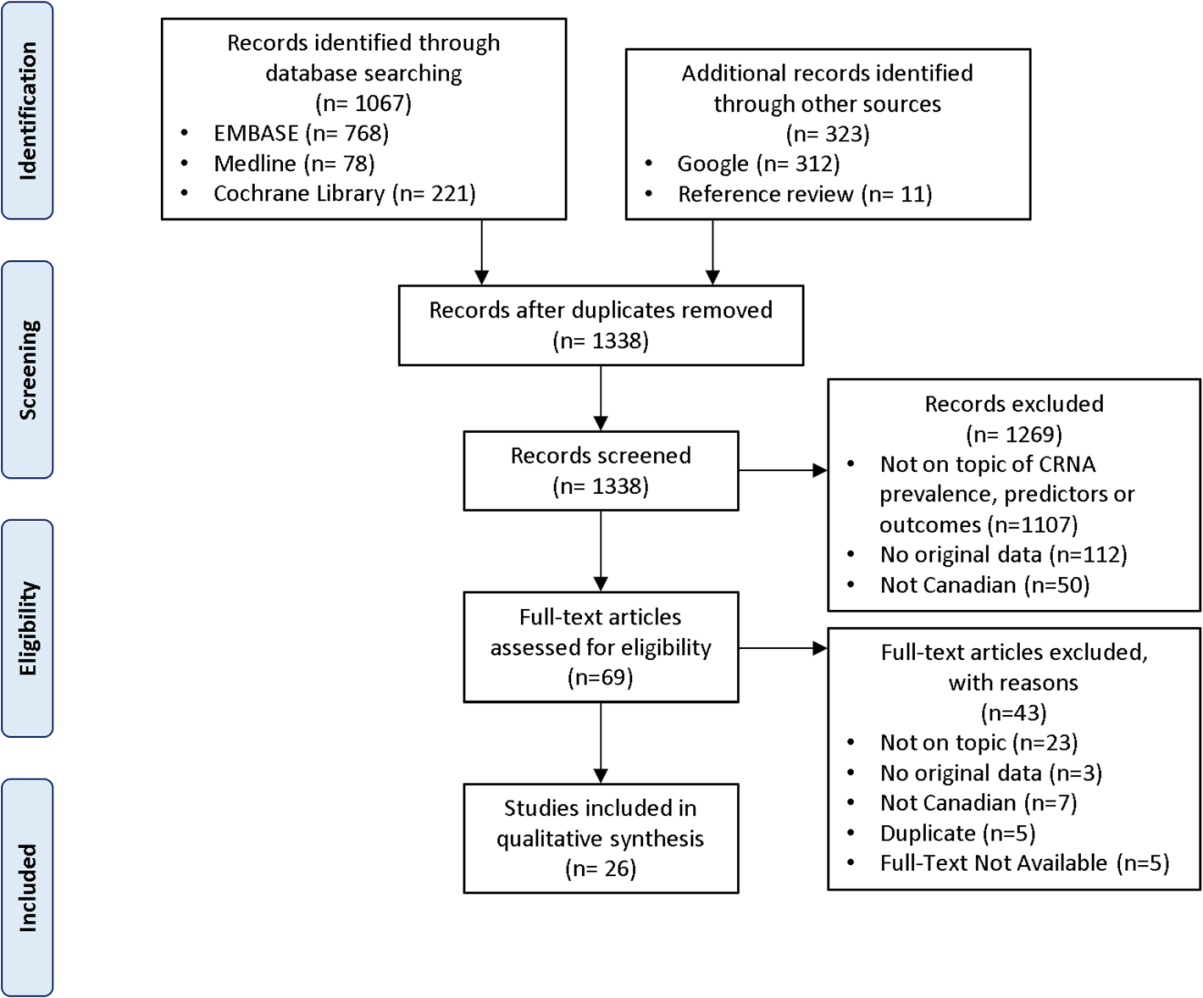
Study flow chart

Of the 1390 articles identified by the literature searches and additional checks, 1321 were excluded based on their titles and abstracts (Fig. 1). Sixty-nine studies were screened in full text with 43 eliminated at this stage, leaving 26 included studies (study details in Table 1) [2, 3, 35–58]. Since several of these studies used the same source survey [3, 38, 39, 43, 45–48], the total sample size of unique participants across all 26 studies is uncertain. Assuming that each study’s participant is a unique individual, the total sample size is 497,534. All but one of the studies were observational, varying from surveys to large healthcare database time series, to qualitative designs. The summary risk of bias was rated as low for eight studies, moderate for nine, and high for nine studies (details in Table 2). All studies reported only on adults, except two studies based on the Canadian Community Health Survey (CCHS) [40, 47] which included those at least 12 years of age. Definitions of CRNA in surveys and the RCT generally included not filling a prescription or skipping doses because of the cost, while the health administrative database studies assumed that declines in utilization shortly after drug policy changes implied CRNA.

**Table 1:** Study characteristics and results

Study ID, design	Demographics	Definition of CRNA	Prevalence of CRNA	Predictors of CRNA^**a**^	Impact on clinical outcomes
Brand 1977 [35] Survey with in-person interviews over a 3-month period, year unspecified	*N* = 225 patients discharged from hospital in Halifax, NS (mean age 57.0)	Not complying with ≥ 1 physician order(s) due to cost of drugs	13.8%	“Cost of drugs” (*p* < 0.001)	N/A
Kennedy 2006 [36] 2002–2003 Joint Canada-US Survey of Health	*N* = 3505 Canadian adults ≥ 18 years	Failure to obtain a prescribed medication due to cost	5.1%	No Canada-specific data	N/A
Hirth 2008 [37] 2002–2004 DOPPS patient questionnaires	*N* = 503 Canadian adult hemodialysis patients from 20 facilities (mean age 62.1, SD 14.7)	Not purchasing medication due to cost	12.9%	Out-of-pocket spending burden (*R*^2^ = 0.44)	N/A
Kennedy 2009 [38] 2007 IHP phone survey	*N* = 2980 Canadian adults ≥ 18 years	Not filling a prescription or skipping doses of medication due to cost during the previous 12 months	8.0%	Younger (< 65 years), multiple chronic conditions, lower household income, each *p* < 0.01 (OR not reported); Quebec (compulsory coverage) compared to Ontario (OR = 0.5, 95% CI 0.3–0.8)	N/A
Kemp 2010 [39] 2007 IHP phone survey	*N* = 2183 Canadian adults ≥ 18 years (median age 50, SE 0.3)	Not filling a prescription or skipping doses of medication due to cost during the previous 12 months	8.0%	Younger age RR = 3.9 (95% CI 2.2–6.9); income below average RR = 3.1 (95% CI 2.1–4.7); high out-of-pocket prescription costs (RR = 4.6 (95% CI 3.8–6.7); first nations RR = 2.1 (95% CI 1.4–3.2); self-reported poor health status RR = 1.5 (95% CI 1.2–2.0); not feeling involved in treatment decisions RR = 1.3 (95% CI 1.1–1.4)	N/A
Law 2012 [40] 2007 CCHS phone survey	*N* = 5732 community-dwelling Canadians ≥ 12 years who received a prescription in the previous year	Altering a prescription to make it last longer or not filling a new prescription or renewing an ongoing prescription, due to cost	Canadian sample, 9.6% (95% CI 8.4–10.7%); QB, 7.2% (4.5–9.8); ON, 9.1% (7.2–11.0%); BC, 17.0% (12.6–21.4%)	Younger age (OR = 4.70, 95% CI 2.91–7.60); low household income (OR = 3.29, 95% CI 2.03–5.33); lack of insurance coverage for drugs (OR = 4.52, 95% CI 3.29–6.20); several chronic health conditions (OR = 1.61, 95% CI 1.07–2.43); fair or poor self-assessed health status (OR = 2.64, 95% CI 1.77–3.94); residing in BC (compared to Ontario) (OR = 2.56, 95% CI 1.49–4.42)	N/A
Zheng 2012 [41] Cross-sectional survey with in-person interviews between March 10 and April 19, 2011	*N* = 60 adult patients attending a general internal medicine rapid assessment outpatient clinic in Hamilton, ON (mean age 60.3, SD 14.3)	Left prescriptions unfilled, delayed filling prescriptions, took prescriptions with reduced frequency or lowered dosages in the previous year because of the cost	15.0%	No drug insurance (OR = 20.7, 95% CI 1.46–292.75); high out-of-pocket expenses (OR = 42.52, 95% CI 2.02–894.03)	N/A
Hunter 2015 [42] HHiT study in-person interviews between January and December 2009	*N* = 716 homeless or vulnerably housed single adults in Vancouver, Toronto, and Ottawa and prescribed ≥ 1 current medication	Not actually taking a current medication prescribed by a doctor as “the medication is too expensive”	3.6%	N/A	N/A
Hennessy 2016 [2] BCPCHC survey between February 2011 and March 2012	*N* = 1849 ≥ 40 year from BC, AB, SK, or MB who reported having heart disease, stroke, diabetes, or hypertension (mean age 65.1, 95% CI 64.3–65.9)	For the previous 12 months, due to cost, either (a) not getting necessary prescription medication or (b) stopping one or more prescribed drug for a week or more	4.1% (95% CI 2.6–6.3%)	Out-of-pocket spending greater than 5% of household income (prevalence RR = 2.6; 95% CI 1.0–6.4)	N/A
Lee 2017 [43] 2014 IHP phone survey	*N* = 4690 community-dwelling Canadians ≥ 55 years	Not filling a prescription or skipping doses within the last 12 months because of out-of-pocket costs	8.3%	QC (compared to ON) (adjusted OR = 0.49, 95% CI 0.29–0.82); younger age (compared to ≥ 65 years): 55–64 years (OR = 3.13, 95% CI 2.27–5.40); poor health status (OR = 1.75, 95% CI 1.12–2.38); low income (OR = 3.59, 95% CI 2.32–5.55); lack of private insurance (OR = 2.33, 95% CI 1.56–3.10)	N/A
Morgan 2017 [3] 2014 IHP phone survey	*N* = 4696 community-dwelling Canadians ≥ 55 years	Not filling a prescription or skipped doses within the last 12 months because of out-of-pocket costs	8.3%	Canadians (compared to the UK) (adjusted OR = 2.25, 95% CI 1.08–4.69); lower income (compared to UK) (OR = 1.23, 95% CI 0.64–2.40)	N/A
Sarnak 2017 [44] OECD data, 2016 IHP phone survey and other sources	*N* = 4547 Canadian adults ≥ 18 years	Not filling/collecting a prescription for medicine or skipped doses because of cost in the past 12 months	Overall: 10.2%; 0 chronic diseases 5.0% vs. 1 chronic disease 12.0% vs. 2+ chronic diseases 16.0%	N/A	N/A
Soril 2017 [45] 2004-14 IHP phone surveys (selected years)	*N* = 25,740 Canadian adults ≥ 18 years	Not filling a prescription because of costs in the previous 12 months	Overall: range 7.1–8.2%; older/sicker adult cohort: range 6.5–19.8%	N/A	N/A
Law 2018 [46] 2016 CCHS phone survey	*N* = 28,091 community-dwelling Canadians ≥ 12 years	Skipping or reducing dosages, or delaying refill prescriptions or not filling prescriptions at all to reduce drug costs	5.5% (95% CI 5.1–6.0%)	Younger adult (*p* < 0.001); out-of-pocket prescription drug spending (*p* < 0.001); lack of drug insurance (*p* < 0.001); lower income (*p* < 0.001); poorer health status (*p* < 0.001)	N/A
Laba 2018 [47] 2016 CCHS phone survey	*N* = 8420 community-dwelling Canadians ≥ 12 years old with ≥ 2 chronic conditions	Skipping or reducing dosages, delaying refill prescriptions, or not filling prescriptions at all to reduce drug costs	10.2% (95% CI 8.6–11.9%); 15.2% (95% CI 11.6–18.8) for respiratory and 16.6% (95% CI 13.2–9.9%) for mental health disorders	Age between 19 and 44 years (OR 2.74, 95%CI 1.76, 4.26); out-of-pocket spending on prescription medicines > CAD500 OR 2.56, 95% CI 1.49, 4.40; lack of drug insurance (OR 3.26, 95% CI 2.12, 4.80); fair to poor health status (OR 3.42, 95% CI 1.46, 8.02); residing in certain provinces, e.g., BC (OR 4.20, 95% CI 2.55, 6.91)	N/A
Men 2019 [48] 2016 CCHS phone survey	*N* = 11,172 community-dwelling Canadians with a prescription within the previous year and answering a food security questionnaire	Skipping or reducing dosages, delaying refill prescriptions, or not filling prescriptions at all to reduce drug costs	8.3%	Household food insecurity adjusted for sociodemographic factors, associated with CRN—RR 1.82 (95% CI 1.00 to 3.31), 3.83 (95% CI 2.44 to 6.03), and 5.05 (95% CI 3.27 to 7.81) for marginally, moderately, and severely food-insecure households, respectively, compared to those with no food insecurity	N/A
Monagle 2018 [49] Phone survey of one anticoagulant clinic	*N* = 110 adult patients newly started on oral anticoagulants in Hamilton, ON	Leaving a prescription unfilled or delaying filling a prescription, or taking less of a medication, due to cost	Warfarin users were more likely to report CRN than NOAC users (40% vs. 13%, *p* = 0.02)	N/A	N/A
Yao 2018 [50] Retrospective pre-post database study 2005–2009 pre- and post-Seniors’ Drug plan policy change (max. out-of-pocket $15 per prescription for patients ≥ 65 years) vs. concurrent control patients 40–64 years not affected by the policy	*N* = 188,109 observed patients in SK	CRNA assumed if adherence post-policy improved compared pre-period and to unaffected control	N/A	Odds of optimal medication adherence: post-SDP (compared to pre-SDP) (OR = 1.08, 95% CI 1.04 to 1.11), but only where OOP costs > $15 per prescription, for prevalent users, for some medication classes. Not compared directly to concurrent control	N/A
Dormuth 2006 [51] Retrospective pre-post database study between June 1997 and 2004 with monthly time series pre- (full coverage) vs. post-policy (copayment)	*N* = 55,752 BC residents ≥ 65 years not in a nursing home, dispensed inhaled corticosteroids (ICS) in 2001 (mean age 75.5)	CRNA assumed if the use of respiratory inhalers declined after policy increasing out-of-pocket expenses	N/A	Initiation of ICS for a new diagnosis of asthma or COPD compared to pre-policy reduced by 25% (95% CI 14–31%); discontinuation of ICS was increased 47% (40–55%) in the copayment group	N/A
Schneeweiss 2007 [52] Retrospective pre-post database study 2000–2004 with repeated measures design, monthly adherence measurement pre- (full coverage) vs. post-policy (copayment)	*N* = 41,561 seniors in BC who were new users of statin drugs	CRNA assumed if use of statins declined after policy increasing out-of-pocket expenses	N/A	Paying 100% out-of-pocket (compared to pre-policy) (OR = 1.94, 95% CI 1.82–2.08); patients post-myocardial infarction or post-revascularization (higher risk) (OR = 0.63, 95% CI 0.59–0.68)	N/A
Schneeweiss 2007 [53] Retrospective pre-post database study 2000–2004 with repeated measures design, monthly adherence measurement pre- (full coverage) vs. post-policy (copayment)	*N* = 13,193 seniors from BC who were new users of β-blockers	CRNA assumed if the use of beta-blockers declined after policy increasing out-of-pocket expenses	N/A	Post-policy cohort (compared to pre-policy) associated with a 1.3% decline in adherence (95% CI 2.5–0.04)	N/A
Goldsmith 2017 [54] Qualitative study with semi-structured interviews of CRNA experience from patients’ perspective 2014–2015	*N* = 35 adults in BC and ON who reported CRNA	Patient self-report of skipping doses, splitting pills, or not filling their prescriptions due to out-of-pocket costs	N/A	Type of insurance; individual’s overall financial flexibility; the burden of drug cost on the individual’s budget; perceived importance of the drug	N/A
Gupta 2019 [55] Qualitative study with semi-structured interviews of strategies used to deal with cost burden	*N* = 12 adult Canadians with spinal cord injuries who reported CRNA	N/A	N/A	Out-of-pocket cost of medication; perceived importance of the drug; lack of drug insurance; competing financial needs, e.g., food, housing; inability to discuss with physicians	N/A
Tamblyn 2001 [56] Retrospective database study with interrupted monthly time series 1993–1997 pre (full coverage for welfare and low-income seniors; $2 copayment for all other seniors) vs. post-policy (25% coinsurance and deductible)	*N* = 70,801 elderly and 25,820 welfare recipients using “essential drugs” in QC	CRNA assumed if post-policy decrease in the use of essential drugs	N/A	Increase in cost sharing associated with a decrease in essential drug use by elderly by 9.1% (95% CI 8.7–9.6) and by welfare recipients by 14.4% (95% CI 13.3–15.6%)	Net increase in serious adverse events by 6.8 and 12.9 per 10,000/month; in ED visits by 14.2 and 54.2 per 10,000/month for elderly and for welfare recipients, respectively
Pilote 2002 [57] Retrospective database study with time-series analysis 1994–1998 pre- (full coverage for welfare and low-income seniors and $2 copayment for all other seniors) vs. post-policy (25% coinsurance and deductible)	*N* = 22,066 patients ≥ 65 years admitted to a QC hospital for a first acute myocardial infarction and discharged alive	CRNA assumed if the proportion of patients who filled at least one prescription during the year after discharge declined post-policy change	N/A	N/A as no change in adherence pre- vs. post-policy	No differences in readmission for cardiac complications, mortality rate, or use of outpatient physician or ED services
Persaud 2019 [58, 59] Randomized open-label trial 2016–2017 with free access including free delivery of prescribed essential medication, compared to usual care	*N* = 786 adults ≥ 18 years old in 9 primary care practices in ON who reported CRNA (mean age 51.7 years, 55.9% female)	Self-reported not filling a prescription or making a prescription last longer because of the cost within the previous 12 months	N/A	No variation in adherence by income	No difference in rates of hospitalization, serious adverse events, or deaths

*CRNA* cost-related nonadherence, *N/A* data not available, *BCPCHC* barriers to care for people with chronic health conditions, *DOPPS* Dialysis Outcomes and Practice Patterns Study, *HHiT* health and housing in transition, *IHP* International Health Policy, *CCHS* Canadian Community Health Survey, *OECD* Organization for Economic Co-operation and Development, *ICS* inhaled corticosteroids, *SDP* Seniors’ Drug Plan, *BC* British Columbia, *AB* Alberta, *SK* Saskatchewan, *MB* Manitoba, *ON* Ontario, *QC* Quebec, *NNT* number needed to treat

^a^Using adjusted or multivariable analyses

**Table 2:**
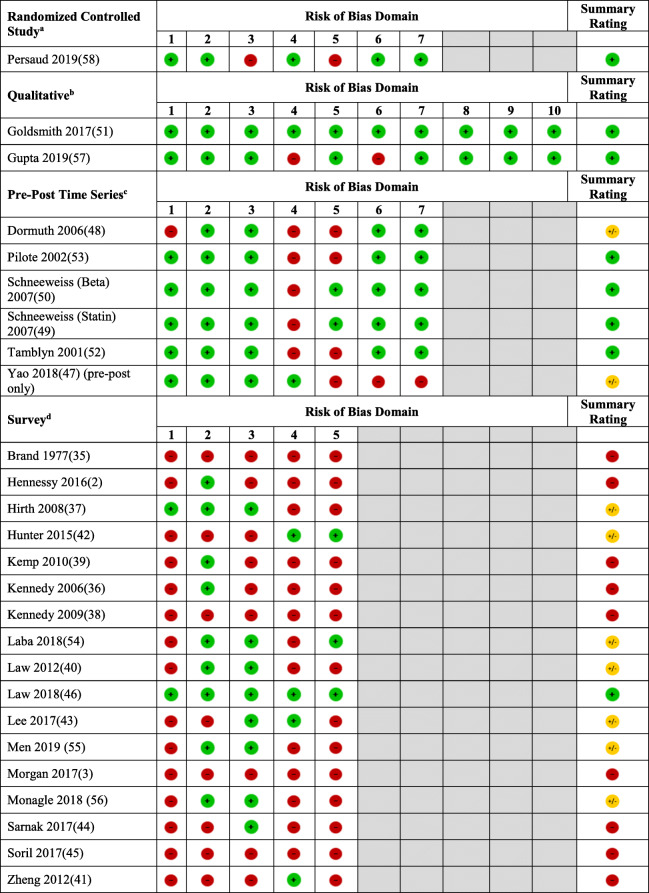
Summary risk of bias ratings

^a^RoB domains for randomized controlled studies (1 = random sequence generation; 2 = allocation concealment; 3 = blinded participants and providers; 4 = blinded outcome assessors; 5 = incomplete outcome data; 6 = selective reporting; 7 = other biases)

^b^RoB domains for qualitative studies (1 = clear statement of aims; 2 = qualitative methods justified; 3 = appropriate design for research aims; 4 = appropriate recruitment strategy; 5 = confidence in data collection; 6 = personal biases; 7 = ethical considerations; 8 = confidence in data analysis; 9 = clear statement of findings; 10 = value of research)

^c^RoB domains for pre-post time series studies (1 = confounding variables/events; 2 = analysis at point of intervention; 3 = intervention effects on data collection; 4 = blinding or objective outcomes; 5 = effect of missing outcome measures; 6 = selective reporting; 7 = other biases)

^d^RoB domains for surveys (1 = representativeness of sample; 2 = adequacy of response rate; 3 = missing data; 4 = pilot testing; 5 = published validity of survey instrument).  denotes a high risk of bias,  denotes a moderate risk of bias, and  denotes a low risk of bias)

### Prevalence of medication CRNA in Canada

Sixteen studies, excluding a medication-specific survey [49], addressed the prevalence of CRNA (*n* = 105,109 potential participants) (Table 1) [2, 3, 35–48]. Using somewhat differing definitions for CRNA and different sampling frames, these studies suggested a prevalence between 3.6 and 15.0% [2, 3, 35–48]. Ten of these studies providing more generalizable and population-level analyses (i.e., not highly selected sub-groups such as the homeless or those with several chronic conditions) based on large national or international surveys suggested rates of 5.1 to 10.2% [3, 36, 38–40, 43–46, 48]. The Joint Canada-US Survey of Health telephone survey in 2002 included 3505 Canadian adults, 5.1% of whom reported CRNA [36]. In the International Health Policy telephone surveys, 8.0% of the sampled Canadian adults reported CRNA in 2007 and 10.2% in 2016 [38, 39, 44]. The CRNA section of the Canadian Community Health Surveys (CCHS) found that 9.6% of adults who received a prescription reported CRNA in 2007 compared to 5.5% overall in 2016 [40, 46]. The 2007 analysis suggested geographic variability, with higher rates of CRNA in British Columbia than other regions [46]. Two studies examined different sub-groups of the 2016 CCHS [47, 48]. Two additional studies estimated CRNA in specific sub-groups groups of Canadian patients, and reported rates of 10.2% in Canadians with comorbidities and 8.3% in participants with food insecurity [37, 41].

### Predictors of CRNA

Nineteen studies (*n* = 440,064 potential participants) provided information on the predictors of CRNA (details in Table 1) [2, 3, 35, 37–41, 43, 46–48, 50–56, 58]. Thirteen studies (*n* = 70,636) analyzed multiple potential factors based on direct reporting from study participants [2, 3, 35, 37–41, 43, 46–48, 54]. Five additional studies (*n* = 369,416) involving large administrative databases used time-series methods with or without pre-post analyses of policies which changed the amount of patient cost-sharing in provinces, to suggest that increased out-of-pocket expenditures for drugs is a predictor of nonadherence assumed to be CRNA [50–53, 56].

Several factors emerged as independent predictors in the studies using multivariable analyses. In order of high to low frequency of mention, these were high out-of-pocket expenses on medication, lower household income or financial flexibility, lack of drug insurance, younger age, poor self-reported health, province of residence, and miscellaneous (Table 3) [2, 3, 35–41, 43, 46–48, 50–57]. The analysis of the CRNA module within the 2007 CCHS was the largest and most detailed, showing a prevalence of 11.4% for the 35 to 44 years age group compared to 4.8% for subjects older than 65 years [40]. In the multivariable analysis, odds ratios were 4.5 for the lack of drug insurance, 3.3 for low household income. 20.1% of participants reporting poor health also reported CRNA compared to 10.4% of subjects reporting good health (OR 2.64, 95% CI 1.77–3.94) [40]. Finally, factors which may reflect differences among jurisdictions including their policies, were also independent predictors. Among those younger than 65 years, respondents in the 2014 International Health Policy Survey (IHPS) who were from Quebec were less likely to report CRNA than those residing in Ontario (OR 0.5, 95% CI 0.3–0.8) [43]. At the time, while drug insurance was compulsory in Quebec, Ontario, reimbursed non-seniors only for those who were socially disadvantaged or had very high medication costs [43]. In the 2007 CCHS, residence in British Columbia where a significant portion of public drug coverage has income-based deductibles was associated with more CRNA compared with Ontario (OR 2.56, 95% CI 1.49–4.42) [40]. The IHPS segment of Canadians self-identifying as First Nations, Inuit or Metis, were at higher risk of CRNA (RR 2.1, 95% CI 1.4–3.2) [39]. Although the publicly funded Non-insured Health Benefits Program includes drug benefits without copayment or deductible, these apply only to those considered “status Indians” or Inuk and require providers to register with the program to avoid initial self-pay [60].

**Table 3:** Predictors of CRNA in Canada

Predictor	No. of articles reporting significance	Citations
Higher out-of-pocket costs^a^	13	[2, 3, 37, 39–41, 47, 50–53, 55, 56]
Lower income or low financial flexibility	9	[3, 38–40, 43, 46, 48, 54, 55]
Lack of drug insurance	7	[40, 41, 43, 46, 47, 54, 55]
Younger age	6	[38–40, 43, 46, 47]
Poor self-reported health status	5	[39, 40, 43, 46, 47]
Province of residence (e.g., Ontario instead of Quebec, or British Columbia instead of Ontario or Quebec)	4	[38, 43, 46, 47]
Several chronic health conditions	2	[38, 46]
High cost of drugs	2	[35, 54]
Low/medium drug importance from individual’s perspective	2	[54, 55]
Not feeling involved in treatment decisions	2	[39, 55]
First Nations status	1	[39]

^a^Includes studies comparing the rates of CRNA pre- and post-copayment policy

Three studies in BC using a similar cohort with similar methodology examined the influence of increased out-of-pocket expense by analyzing the effect of changes in drug insurance coverage on adherence measured by prescription dispensing intervals [51–53]. The utilization of maintenance respiratory inhalers declined by approximately 5.8 to 12.3% (*p* < 0.001), the rate of full adherence to statins decreased by 5.4% (95% CI, 6.4 to 4.4%), but adherence to beta-blockers was only modestly reduced (approximately 1%) compared to full coverage [51–53]. Nonadherence was associated with higher out-of-pocket expenditures, with beta-blockers thought to be less affected because of their low cost compared to the other drug groups at the time of the study [53]. For statins, adherence was better in high-risk patients with prior vascular events compared to the entire group [52]. An analysis of a policy change to lower seniors’ out-of-pocket prescription drug costs in Saskatchewan in 2007, found a small increase in optimal medication adherence after the policy change [50].

#### CRNA association with clinical outcomes

Only three studies measured clinical outcomes potentially related to CRNA (Table 1; *n* = 93,653) [56–58]. The highest quality study was a recent randomized controlled trial involving patients in primary care in Ontario who reported that they did not fill a prescription or changed regimens to make their supply last longer because of the cost. The study found that the intervention group provided free, mailed prescriptions deemed essential, reported better adherence, improved perceived care, and less concern about making ends meet at 12 months follow-up. Several surrogate outcomes were followed, with improvement in blood pressure in the intervention group for those requiring anti-hypertensives but no significant improvement in A1C or cholesterol. However, there was no difference in hospitalizations, serious adverse events, or death.

The introduction of a drug policy in Quebec in the nineties increased out-of-pocket costs for all residents. In one retrospective study, this led to a decrease in the overall number of drugs used per day by the elderly and by welfare recipients, including “essential” medications such as aspirin and furosemide (decrease of 9.1–14.4%) as well as symptomatic but potentially harmful drugs such as benzodiazepines (decrease of 15.1–22.4%). The decline in the use of essential drugs was associated with a small increase in serious adverse events including death, hospital or nursing home admission, or emergency department visits [56]. In a second retrospective study, there was no change in adherence to post-myocardial infarction medication adherence and no change in clinical outcomes after the policy compared to pre-policy [57].

## Discussion

We believe that this is the first systematic review to focus on the relationship between medication costs and medication adherence in Canada. All but one of the studies in our review were observational therefore susceptible to bias and confounders. We found rates of CRNA range from 5.1 to 10.2% in general surveys of the population over time, suggesting that an important minority of the population is experiencing problems with prescription medication adherence due to their medication cost. The range is likely explained by differing sampling frames, questions, definitions of CRNA, and statistical uncertainty. The international studies in our review suggest that Canadian rates of CRNA are in the middle of other developed countries. In the IHPS survey, the rate of CRNA in Canada (8%) was in the middle of seven countries, with the Netherlands having the lowest rate (3%) and the USA having the highest rate (20%) [39]. In the dialysis study, the rate of CRNA in Canada (12.9%) was similar to the overall rate of CRNA among 12 countries (13.4%), with Japan being the lowest rate (3.2%) and the USA being the highest rate (29.2%) [38].

Overall, predictors for CRNA in Canada revolved around lack of affordability, younger age, chronic illness, private insurance coverage, and province of residence. This likely reflects the characteristics of the different public drug plan coverage programs and different financial capability to afford medicines in different provinces. None of the studies developed or used a clinical prediction rule, which would examine the risk factors together to determine how their quantitative combination influences risk [61]. This is a well-established method to refine population risk to individual risk. Both qualitative studies found that patients weighed their financial obligations against the perceived importance of the medications in making their adherence decisions and recognized that they sometimes were making decisions that might adversely affect their health [54].

The lack of current information on the association of CRNA with clinical outcomes in Canada is very troubling, as this is the primary question of interest both for clinicians and policymakers. Although low adherence to beneficial medications has previously been linked to increased mortality, the data may be biased due to the “healthy user” effect [17]. Randomized trials show that interventions to improve adherence do so only modestly and do not seem to improve patient outcomes [62]. Two recent randomized controlled trials (RCTs) in the USA directly address whether removing medication cost improves clinical outcomes. The aforementioned MI FREEE RCT found that free coverage for essential cardiovascular medications post-myocardial infarction increased adherence by 4 to 6% (*p* < 0.001) but did not improve the primary outcome of the first major vascular event or procedure [20]. More recently, the ARTEMIS trial also found that provision of free access to P2Y_12_ inhibiting anti-platelet agents for a year increased adherence by a small amount (2.3%), but there was no difference in major adverse cardiovascular events [63]. In addition, since patients are frequently taking medications that are not essential and may be harmful, decreased adherence to these medications may not lead to adverse outcomes. Two of our studies suggested that participants reported increased health care utilization as a result of their CRNA, but did not actually measure clinical outcomes or healthcare utilization [46, 55]. The sole RCT in our SR found that the free provision and delivery of essential medications increased adherence by 10% and improved one of three clinical surrogates at 12 months follow-up, but did not improve clinical outcomes [58]. In summary, the relationship between medication costs, medication adherence, and patient outcomes is more complex than originally thought.

This systematic review has limitations worth noting. First, since studies varied in their methods of measurement, quantitative pooling was not possible. Second, there is no gold standard measure for medication adherence, so there are likely measurement errors with each of the methods used. Third, questionnaire studies are susceptible to responder and recall bias, and the studies examining adherence before and after policy changes are somewhat indirect inferences regarding the impact of costs. Fourth, we were unable to find information on how different types of insurance—co-pays, deductibles, annual maximums, etc.—influence the prevalence of CRNA. Finally, since multiple behavioral attributes are associated with nonadherence, it would take a very large prospective study to determine the specific impact of medication cost on adherence.

The findings of this systematic review have several implications. First, as CRNA may affect a large number of Canadians, communication between providers and patients regarding affordability of prescribed medications is essential and may play an important role in the reduction of CRNA. Second, the evidence summarized here will be useful to inform the debate on a national PharmaCare program where proponents cite estimates of higher health care utilization because of the patient burden of medication costs while opponents cite the lack of evidence that removal of patient-borne costs improves outcomes [64, 65]. Modeling of a universal drug benefit program would benefit from better estimates of the impact of CRNA on health care utilization and clinical outcomes [28]. The association of high out-of-pocket medication costs with lower adherence might argue for improved drug coverage for those with low incomes. However, the high-quality evidence so far suggests that more research is required to determine for which people, which drugs, which situations, and how much cost relief might be required to improve clinical outcomes

## Conclusion

Our systematic review suggests that an important minority of Canadians may not be adherent to medications because of their costs. Financial factors appear to be the main predictors of CRNA, suggesting that drug program design and coverage have a significant influence on CRNA rates. However, consistent with international evidence to date, removal of all medication cost for essential drugs for patients with CRNA has not been shown to improve clinical outcomes.

## Supplementary Information

**Additional file 1.** PRISMA 2009 Checklist for CRN SR Canada.

**Additional file 2.** Appendix. MEDLINE Search Strategy.

## Data Availability

All data generated or analyzed during this study are included in this published article and its supplementary information files.

## Abbreviations

CRNA: Cost-related nonadherence
ED: Emergency department
CCHS: Canadian Community Health Survey
IHPS: International Health Policy Survey
