# Supplementary material for: Cost-related medication nonadherence in Canada: a systematic review of prevalence, predictors, and clinical impact
Source: Syst Rev. 2021 Jan 6;10:11. doi: 10.1186/s13643-020-01558-5 (PMC7788798; doi:10.1186/s13643-020-01558-5)
Supplement: Supplementary file 2 — Additional file 2. Appendix. MEDLINE Search Strategy. [file 13643_2020_1558_MOESM2_ESM.pdf]

APPENDIX 1. MEDLINE Search Strategy

Database: OVID Medline Epub Ahead of Print, In-Process & Other Non-Indexed Citations, Ovid MEDLINE(R) Daily and Ovid MEDLINE(R) 1946 to Present
Search Strategy:
----
1 *Medication Adherence/ (8545)
2 Patient Compliance/ (54949)
3 persisten$.mp. (286317)
4 adheren$.mp. (159735)
5 complian$.mp. (157679)
6 1 or 2 or 3 or 4 or 5 (572582)
7 Drug Costs/ (14578)
8 Prescription Fees/ (1123)
9 Fees, Pharmaceutical/ (1269)
10 cost sharing.mp. (3230)
11 co-pay.mp. (78)
12 "Deductibles and Coinsurance"/ (1631)
13 self-pay.mp. (575)
14 out-of-pocket.mp. (4212)
15 Insurance Coverage/ (11433)
16 7 or 8 or 9 or 10 or 11 or 12 or 13 or 14 or 15 (34851)
17 exp Canada/ (145847)
18 canadian$.mp. (52774)
19 (British Columbia$ or alberta$ or saskatchewan$ or manitoba$).mp. [mp=title, abstract, original title, name of substance word, subject heading word, keyword heading word, protocol supplementary concept word, rare disease supplementary concept word, unique identifier, synonyms] (31518)
20 (ontari$ or quebec$ or new brunswick$ or nova scotia$).mp. [mp=title, abstract, original title, name of substance word, subject heading word, keyword heading word, protocol supplementary concept word, rare disease supplementary concept word, unique identifier, synonyms] (59631)
21 (prince edward island$ or newfoundland$ or yukon$ or northwest territor$ or nunavut$).mp. [mp=title, abstract, original title, name of substance word, subject heading word, keyword heading word, protocol supplementary concept word, rare disease supplementary concept word, unique identifier, synonyms] (4260)
22 17 or 18 or 19 or 20 or 21 (188579)
23 6 and 16 and 22 (66)
****
